# Blood Donation Practice and Associated Factors in Ethiopia: A Systematic Review and Meta-analysis

**DOI:** 10.1155/2020/8852342

**Published:** 2020-11-25

**Authors:** Addisu Getie, Adam Wondmieneh, Melaku Bimerew, Getnet Gedefaw, Asmamaw Demis

**Affiliations:** ^1^Department of Nursing, College of Health Sciences, Woldia University, P.O. Box 400, Woldia, Ethiopia; ^2^Department of Midwifery, College of Health Sciences, Woldia University, P.O. Box 400, Woldia, Ethiopia

## Abstract

**Background:**

Blood donation is a novel act to save the lives of people who face serious medical and surgical conditions. Since the demand for blood supply is too high, there is a shortage of blood which causes significant morbidity and mortality. To increase blood supply and maintain adequate quantity of blood, regular and volunteer blood donation practice is needed, which meets the increased demand for blood. Therefore, this systematic review and meta-analysis was aimed at assessing the prevalence of blood donation practices and associated factors in Ethiopia.

**Method:**

PubMed/MEDLINE, HINARI, Embase, Scopus, Google Scholar, African Journals Online (AJOL), and published and unpublished articles from the Ethiopian University repository were searched to find articles. Cochrane *I*^2^ statistics and Egger's test with funnel plots were done to check heterogeneity and publication bias, respectively. Subgroup analysis by region, study subjects, study setting, and sample size was done due to heterogeneity, as well as sensitivity analysis.

**Result:**

Twenty studies from different regions with a total study subject of 8546 were included in the final review. The pooled prevalence of blood donation practice in Ethiopia was 25.82% (95% CI: 21.45-30.19). Having good knowledge of blood donation (AOR = 2.85; 95% CI: 2.33-3.48) and favorable attitude (AOR = 4.35; 95% CI: 2.93-6.45) were factors associated with blood donation practice in Ethiopia.

**Conclusion:**

The pooled prevalence of blood donation practices in Ethiopia was short of the demand for blood due to the increase in serious medical conditions and road traffic accidents. Knowledge and attitude towards blood donation were significantly associated with blood donation practice. Therefore, awareness creation and health education programs targeting blood donation practice should be strengthened.

## 1. Introduction

Blood is a special type of body fluid with special components that deliver essential substances like oxygen and nutrients to the body's cells and used for the transportation of different metabolic wastes [1]. Blood donation is the process of collecting blood from voluntary donors who are at low risk for any infection and unlikely to jeopardize their health by blood donation [2]. It is a lifesaving practice for people who face blood loss due to road traffic accidents, surgery, pregnancy complications, chemotherapy, and diseases like malaria, anemia, and intestinal parasites which increase the demand for blood [3]. Despite an extensive effort and many blood donation programs, still there is a shortage of safe blood to meet the demands in sub-Saharan counties due to an increased prevalence of anemia and malaria [4, 5]. From an estimated 80,000,000 units of blood donated annually worldwide, only 38% is collected in the developing countries where over 80% of populations live [6].

Studies conducted in Asia showed that the prevalence of blood donation practices was 10%, 18%, 23%, and 35.69% [7–10]. According to studies done in Africa, the prevalence of blood donation ranges from 10.64% to 61.69% in which the majority of blood donation practice prevalence lays lower than 30% [11–16]. Studies in different parts of the world identify factors that hinder blood donation practice. Such perceptions include being not fitted to give blood, fear of anemia, fear of different health risks, and lack of information about blood donation, which result in the significant morbidity and mortality [17].

Therefore, the suitability of prospective voluntary and regular blood donors worldwide is essential to protect the safety and sufficiency of blood supply and safeguard the health of blood recipients [2]. Even if different studies were done, the pooled prevalence and associated factors of blood donation practice in Ethiopia are not well known. Hence, the purpose of this review is to assess the prevalence of blood donation practices and associated factors in Ethiopia.

## 2. Methods and Materials

### 2.1. Study Protocol

In this systematic review and meta-analysis, the Preferred Reporting Items for Systematic Review and Meta-analysis (PRISMA) were used for the reporting of findings [18] (Table S1).

### 2.2. Databases and Search Strategy

The searched databases include PubMed/MEDLINE, HINARI, Embase, Scopus, Google Scholar, and African Journals Online (AJOL). The published and unpublished articles were also searched from Ethiopian university repositories. The articles were searched from January 1, 2000, up to August 15, 2020. Then, research articles published until August 15, 2020, were included in the final analysis. In this review, articles reporting the prevalence of blood donation practices and its associated factors in Ethiopia were included. The search items used were as follows: “blood donation”, “volunteer blood donation” , “blood donation practice”, “volunteer blood donation practice” , “factors”, “associated factors”, “determinant factors”, “university students”, “residents”, “health care workers” , “health care providers” , “health professionals”, and “Ethiopia”. The search strings were developed using “AND” and “OR” Boolean operators (Table S2).

### 2.3. Searching and Eligibility Studies

The retrieved studies were exported into “EndNote reference software version 8 (Thomson, Stamford, CT, USA) citation manager” to sort and avoid duplication of articles. Two investigators (AG and MB) independently evaluated each article by title and abstract using inclusion criteria and assessed the eligibility of the articles for the final analysis. In the extraction sheet, the first name of authors, publication year, the region where the study was conducted, study setting, study subjects, method of survey, study period, sample size, study design, level of education, professional category, the prevalence of blood donation practice, and factors associated with blood donation practice were extracted.

### 2.4. Inclusion and Exclusion Criteria

Both published and unpublished observational studies reporting the prevalence of blood donation practices in Ethiopia were included. Articles reported other than the English language, case reports, review articles, trials, updates, news, qualitative studies, articles not reporting the outcome of the study, and studies without full text after contacting the corresponding author were excluded.

### 2.5. Outcome Measurement of the Study

This systematic review and meta-analysis has two major outcomes: (1) blood donation practice which is measured by the experience of blood donation at least once in their lifetime and (2) the factors associated with blood donation practice.

### 2.6. Quality Assessment

Two independent authors (AG and AD) assessed the quality of the studies using the Newcastle Ottawa Scale (NOS) for cross-sectional studies [19]. All articles included in the study were cross-sectional by design. The methodological quality of the study, comparability of the study, and the outcome and statistical analysis of the study were the three major assessment tools that we used to declare the quality of the study. Lastly, studies that scored a scale of ≥7 out of 10 was considered achieving high quality. During quality appraisal of the articles, any discrepancies between the two authors were resolved. All the studies were included based on the Newcastle Ottawa Scale quality assessment criteria. All authors independently assessed the articles for consideration and inclusion for the study.

### 2.7. Data Processing and Analysis

The overall prevalence of blood donation practice and factors associated with blood donation practice were pooled using a weighted inverse variance random-effects model at 95% Cl [20]. The data were extracted using Microsoft Excel spreadsheets. Then, the data were exported to STATA version 11 statistical software for analysis. The Cochrane Q-test and *I*^2^ with its corresponding *P* value were used to assess the heterogeneity of the study. Low, moderate, and high heterogeneity are represented by the following values of *I*^2^, respectively: 25%, 50%, and 75% [21]. The source of heterogeneity was examined through subgroup analysis based on (region, study subjects, study setting, and sample size). Sensitivity analysis was also executed to confirm the presence or absence of influential studies observed in the pooled prevalence of blood donation practices and associated factors in Ethiopia. The presence of publication bias was evaluated by using Egger's test and presented with funnel plots [22]. For association, a log odds ratio was used to decide the association between associated factors and blood donation practice. A statistical test with a *P* value of less than 0.05 was considered statistically significant.

## 3. Result

**Figure 1:**
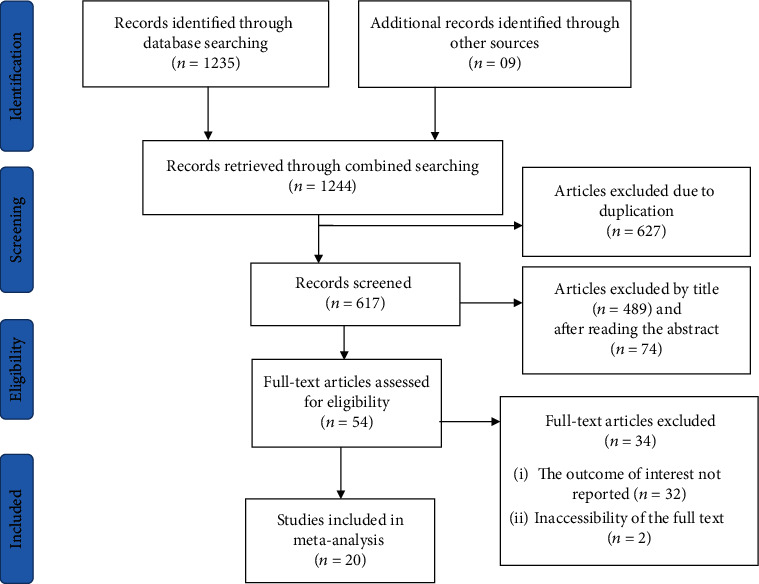
Flow chart of selection for systematic review and meta-analysis of blood donation practice and associated factors in Ethiopia.

We retrieved a total of 1240 articles from the PubMed/MEDLINE, HINARI, Embase, Google Scholar, and African Journals Online (AJOL) databases and from Ethiopian university repositories about blood donation practices and associated factors in Ethiopia. Out of all the retrieved articles, only 20 articles were left for systematic review and meta-analysis with the final sum of 8546 study participants (Figure 1).

### 3.1. Characteristics of the Studies and Study Participants

Twenty articles were included in this systematic review and meta-analysis: 7 from the Amhara region [11, 13, 17, 23–26], 6 from the Oromia region [6, 27–31], 2 from the Addis Ababa city administration [12, 14], 2 from the Southern Nation Nationalities and Peoples Region (SNNPR) [32, 33], 2 from the Tigray region [34, 35], and 1 from the Afar region [4]. There are 8546 study participants (4860 male and 3686 female) included in this review. All studies were cross-sectional in design, and the sample size of the included studies ranged from 218 to 845 (Table S3).

### 3.2. Blood Donation Practice in Ethiopia

**Figure 2:**
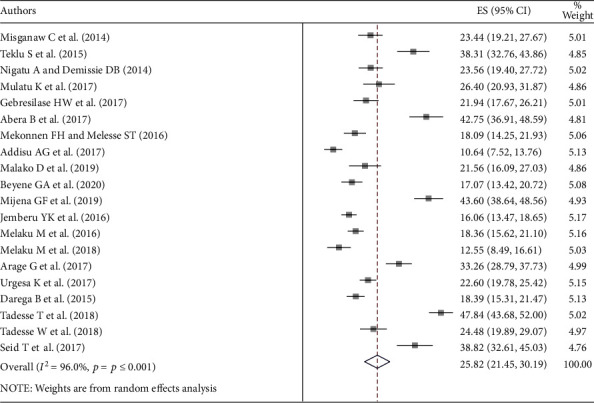
Forest plot of the pooled prevalence of blood donation practice in Ethiopia.

The overall pooled prevalence of blood donation practice in Ethiopia was 25.82% (95% CI: 21.45-30.19) (Figure 2).

### 3.3. Heterogeneity and Publication Bias

In this systematic review and meta-analysis, heterogeneity was detected within the studies (*I*^2^ = 96.0%, *P* ≤ 0.001). The funnel plot showed that there is asymmetrical distribution of studies included in the review, and Egger's test was statistically significant (*P* = 0.002) suggesting the presence of publication bias (Figure S1).

### 3.4. Subgroup Analysis

Subgroup analysis was done based on the region in which the study was conducted, study subjects, study setting, method of survey, and sample size. Accordingly, the highest pooled prevalence of blood donation practice was reported in Addis Ababa city administration with 37.05% (95% CI: 22.23-51.85). Similarly, the highest prevalence of blood donation practice was reported on studies having health care workers as a study subject: 37.12% (95% CI: 29.61-44.63). The prevalence of blood donation practice is higher on studies conducted in institution-based cross-sectional studies: 28.81% (95% CI: 22.57-35.05), than community-based cross-sectional studies: 21.39% (95% CI: 16.00-26.77). Studies with the self-administered method of the survey have a higher prevalence of blood donation practice: 29.95% (95% CI: 23.73-36.17) (Table 1).

Other∗: Tigray and Afar. SNNPR: South Nation Nationalities and Peoples Region.

### 3.5. Sensitivity Analysis

In this systematic review and meta-analysis, a leave one-point sensitivity analysis conducted using the random-effects model suggested that none of the points estimate outside of the overall 95% confidence interval, confirming that there is no influential study (Table S4).

### 3.6. Factors associated with Blood Donation Practice in Ethiopia

**Figure 3:**
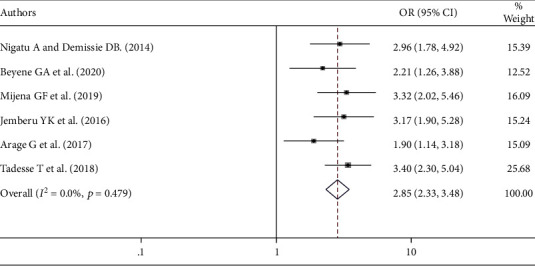
The overall pooled odds ratio of the association between knowledge and blood donation practice in Ethiopia.

In this systematic review and meta-analysis, there is a significant association between knowledge about blood donation and blood donation practice. Being knowledgeable about blood donation was 2.85 times more likely to have blood donation in practice than their counterparts (AOR = 2.85; 95% CI: 2.33-3.48) (Figure 3).

**Figure 4:**
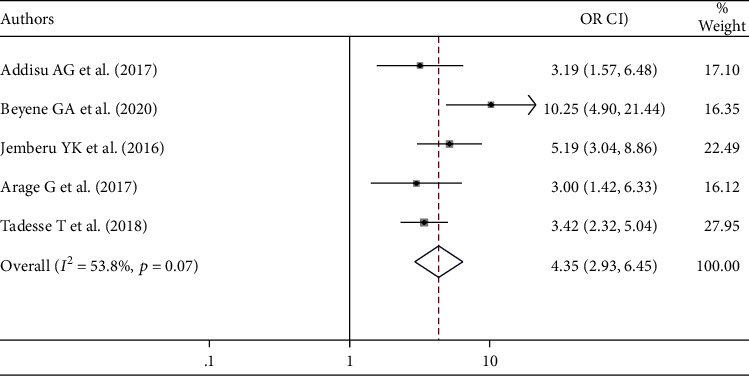
The overall pooled odds ratio of the association between attitude and blood donation practice in Ethiopia.

This review also showed that there is an association between attitude and blood donation practice. Individuals having favorable attitude were 4.35 times more likely to donate blood than those who had unfavorable attitude (Figure 4).

## 4. Discussion

Blood donation practice is part of a lifesaving activity that millions need during different emergencies and medical conditions. The availability of safe blood is highly needed due to an increase in conditions like cancer, anemia, malaria, pregnancy-related conditions, and road traffic accidents. Therefore, this systematic review and meta-analysis was aimed at assessing the prevalence of blood donation practice and its associated factors in Ethiopia. In this review, the pooled prevalence of blood donation practice in Ethiopia was 25.82% (95% CI: 21.45-30.19). This result is not enough to address the safe amount of blood to those who need blood in Ethiopia due to an increased different medical and other condition that needs blood to continue life. The reason might be due to poor awareness and an unfavorable attitude towards blood donation practice. The prevalence of blood donation practice in this review was lower than studies done in Saudi (58.2%) [36], Malaysia (35.6%) [9], and Brazil (32%) [37]. This variation might be due to the difference in the knowledge and willingness of the study subjects towards blood donation practice. It might be also due to the variation of media coverage and the educational status of the peoples. The result of this study is in line with a study conducted in Iran (26%) [38].

The prevalence of blood donation practice in Nigeria and India was 15% and 23%, respectively [8, 16]. These values are lower than those found in the current study. This discrepancy might be due to the difference in need of blood to be donated, wherein the need for blood is too high in Sub-Saharan countries, including Ethiopia, which motives the blood donors.

In this systematic review and meta-analysis, the heterogeneity of the studies does not influence the overall result. The quality of the studies was assessed by Newcastle Ottawa Scale with the following components: representative of the sample, sample size, nonrespondent, ascertainment of the exposure, comparability, and outcome. By considering these components, the quality of the included studies scored seven and above in Newcastle Ottawa Scale. Of all included studies, nine scored seven, six scored eight, and the remaining five scored nine out of ten. The study also showed that there was a publication bias. Then, sensitivity analysis and subgroup analysis were done to overcome the presence of publication bias.

In the subgroup analysis, there is a variation of blood donation practice within regions, study participants, study setting, method of survey, and sample size category. Regarding region, the highest prevalence of blood donation practice was reported in Addis Ababa city administration and the lowest was reported in the Amhara region. The reason might be due to the difference in information access wherein Addis Ababa is more urbanized than the others. As a result, there is high media coverage and the presence of blood donation centers. Of all respondents, the prevalence of blood donation practice is higher in health care workers than in university students and communities. This is due to the fact that health care workers are more knowledgeable and had a favorable attitude due to educational access and frequent exposure to individuals who need blood. Thus, these conditions motive them to donate blood.

This review showed that the prevalence of blood donation practice of study subjects from institutions (universities, hospitals, and health centers) was higher than those of study subjects from out-of institutions. The possible reason might be that institutions are the source of knowledge and information. Therefore, study subjects from institutions had different factors that motivate them to practice blood donation.

Having good knowledge about blood donation was 2.85 times more likely to practice blood donation than having poor knowledge. This finding is supported by studies done in Saudi Arabia [36], North India [39], and Pakistan [40]. This might be due to the fact that knowledgeable individuals regarding blood donation know more about the essentiality of blood safety in saving the lives of patients who need blood and have different motive factors to donate blood. The odds of blood donation practice were 4.35 times more likely among individuals having a favorable attitude than those who had an unfavorable attitude towards blood donation practice. This finding is supported by the studies conducted in Brazil [37] and Benin [41]. The possible reason is due to the fact that individuals having a favorable attitude are more willing to donate blood.

## 5. Conclusion

The overall pooled prevalence of blood donation practices was low in relation to the demand for blood in Ethiopia due to the increase in serious medical conditions and road traffic accidents. The knowledge of individuals about blood donation practice and their attitude towards blood donation practice was significantly associated factors with blood donation practice in Ethiopia. Therefore, the authors recommend that there should be a creation of awareness through different campaigns and avail information access through mass Media.

### 5.1. Strength and Limitation of the Study

This study covers a wide area and investigates different articles making the review more accurate, and subgroup and sensitivity analyses were done which addresses heterogeneity checking for related existing influential studies. However, almost all included studies were cross-sectional in deign which may limit to generate a desired cause and effect link.

## Figures and Tables

**Table 1:** Subgroup analysis of blood donation practice and associated factors in Ethiopia (*n* = 20).

Variables	Subgroup	Studies (*n*)	Population	Prevalence (95% CI)	*I* ^2^ (%)	*P* value
Region	Amhara	7	3261	21.41 (14.88-27.94)	95.8	*P* < 0.001
	Oromia	6	3006	24.36 (18.16-30.75)	94.1	*P* < 0.001
	Addis Ababa	2	679	37.05 (22.23-51.85)	94.1	*P* < 0.001
	SNNPR	2	468	23.98 (19.24-28.72)	33.5	0.220
	Other∗	3	1132	30.76 (16.19-45.33)	96.4	*P* < 0.001
Study participants	Health care workers	6	2009	37.12 (29.61-44.63)	92.1	*P* < 0.001
	Community	8	4191	21.39 (16.00-26.77)	95.1	*P* < 0.001
	University students	6	2346	20.63 (17.07-24.20)	78.9	*P* < 0.001
Study setting	Institution based	11	3746	28.81 (22.57-35.05)	95.7	*P* < 0.001
	Community based	9	4800	21.39 (16.00-26.77)	95.1	*P* < 0.001
Survey method	Self-administered	13	4738	29.95 (23.73-36.17)	96.0	*P* < 0.001
	Face to face interview	7	3808	18.22 (14.91-21.53)	86.0	*P* < 0.001
Sample size	<400	13	4159	26.47 (20.62-32.32)	95.4	*P* < 0.001
	≥400	7	4387	24.69 (17.47-3191)	97.2	*P* < 0.001

## Data Availability

All related data have been presented within the manuscript. The dataset supporting the conclusions of this article is available from the authors on request.

## Abbreviations

AJO: African Journals Online
AOR: Adjusted odds ratio
CI: Confidence interval
NOS: Newcastle Ottawa Scale
PRISMA: Preferred Reporting Items for Systematic Review and Meta-analysis
SNNP: South Nation Nationalities and Peoples.

## Supplementary Materials

Supplementary Materials Figure S1: funnel plot with 95% confidence limits of the pooled prevalence of blood donation practice in Ethiopia. Table S1: Preferred Reporting Items for Systematic Review and Meta-analysis guideline for reporting of the findings. Table S2: searches for the MEDLINE/PubMed and Google Scholar databases for blood donation practice and associated factors in Ethiopia. Table S3: study characteristics included in the review and meta-analysis of blood donation practices and associated factors in Ethiopia. Table S4: Sensitivity analysis on blood donation practice and associated factors in Ethiopia.
