# Supplementary material for: Blood Donation Practice and Associated Factors in Ethiopia: A Systematic Review and Meta-analysis
Source: Biomed Res Int. 2020 Nov 25;2020:8852342. doi: 10.1155/2020/8852342 (PMC7714580; doi:10.1155/2020/8852342)
Supplement: Supplementary Materials — Figure S1: funnel plot with 95% confidence limits of the pooled prevalence of blood donation practice in Ethiopia. Table S1: Preferred Reporting Items for Systematic Review and Meta-analysis guideline for reporting of the findings. Table S2: searches for the MEDLINE/PubMed and Google Scholar databases for blood donation practice and associated factors in Ethiopia. Table S3: study characteristics included in the review and meta-analysis of blood donation practices and associated factors in Ethiopia. Table S4: Sensitivity analysis on blood donation practice and associated factors in Ethiopia. [file 8852342.f1.pdf]

Table s1: Preferred Reporting Items for Systematic Review and Meta-analysis guideline for reporting of the findings

Section/topic	#	Checklist item	Reported on page #
**TITLE**			1
Title	1	Identify the report as a systematic review, meta-analysis, or both.	1
**ABSTRACT**			2
Structured summary	2	Provide a structured summary including, as applicable: background; objectives; data sources; study eligibility criteria, participants, and interventions; study appraisal and synthesis methods; results; limitations; conclusions and implications of key findings; systematic review registration number.	2
**INTRODUCTION**			3
Rationale	3	Describe the rationale for the review in the context of what is already known.	3
Objectives	4	Provide an explicit statement of questions being addressed with reference to participants, interventions, comparisons, outcomes, and study design (PICOS).	3
**METHODS**			4
Protocol and registration	5	Indicate if a review protocol exists, if and where it can be accessed (e.g., Web address), and, if available, provide registration information including registration number.	NA
Eligibility criteria	6	Specify study characteristics (e.g., PICOS, length of follow-up) and report characteristics (e.g., years considered, language, publication status) used as criteria for eligibility, giving rationale.	5
Information sources	7	Describe all information sources (e.g., databases with dates of coverage, contact with study authors to identify additional studies) in the search and date last searched.	4
Search	8	Present full electronic search strategy for at least one database, including any limits used, such that it could be repeated.	4
Study selection	9	State the process for selecting studies (i.e., screening, eligibility, included in systematic review, and, if applicable, included in the meta-analysis).	5
Data collection process	10	Describe method of data extraction from reports (e.g., piloted forms, independently, in duplicate) and any processes for obtaining and confirming data from investigators.	5
Data items	11	List and define all variables for which data were sought (e.g., PICOS, funding sources) and any assumptions and simplifications made.	5
Risk of bias in individual studies	12	Describe methods used for assessing risk of bias of individual studies (including specification of whether this was done at the study or outcome level), and how this information is to be used in any data synthesis.	6
Summary measures	13	State the principal summary measures (e.g., risk ratio, difference in means).	6
Synthesis of results	14	Describe the methods of handling data and combining results of studies, if done, including measures of consistency (e.g., I^2) for each meta-analysis.	6

Section/topic	#	Checklist item	Reported on page #
Risk of bias across studies	15	Specify any assessment of risk of bias that may affect the cumulative evidence (e.g., publication bias, selective reporting within studies).	6
Additional analyses	16	Describe methods of additional analyses (e.g., sensitivity or subgroup analyses, meta-regression), if done, indicating which were pre-specified.	6
**RESULTS**			7
Study selection	17	Give numbers of studies screened, assessed for eligibility, and included in the review, with reasons for exclusions at each stage, ideally with a flow diagram.	7
Study characteristics	18	For each study, present characteristics for which data were extracted (e.g., study size, PICOS, follow-up period) and provide the citations.	8
Risk of bias within studies	19	Present data on risk of bias of each study and, if available, any outcome level assessment (see item 12).	10
Results of individual studies	20	For all outcomes considered (benefits or harms), present, for each study: (a) simple summary data for each intervention group (b) effect estimates and confidence intervals, ideally with a forest plot.	9
Synthesis of results	21	Present results of each meta-analysis done, including confidence intervals and measures of consistency.	10
Risk of bias across studies	22	Present results of any assessment of risk of bias across studies (see Item 15).	11
Additional analysis	23	Give results of additional analyses, if done (e.g., sensitivity or subgroup analyses, meta-regression [see Item 16]).	12
**DISCUSSION**			14
Summary of evidence	24	Summarize the main findings including the strength of evidence for each main outcome; consider their relevance to key groups (e.g., healthcare providers, users, and policy makers).	14
Limitations	25	Discuss limitations at study and outcome level (e.g., risk of bias), and at review-level (e.g., incomplete retrieval of identified research, reporting bias).	16
Conclusions	26	Provide a general interpretation of the results in the context of other evidence, and implications for future research.	16
**FUNDING**			NA
Funding	27	Describe sources of funding for the systematic review and other support (e.g., supply of data); role of funders for the systematic review.	NA

Table S2: Searches for the MEDLINE/ PubMed and Google Scholar databases for blood donation practice and associated factors in Ethiopia.

Databases	Searching terms	Number of studies
MEDLINE/ PubMed	“blood donation” OR “blood donation practice” AND "factors" OR “associated factors” OR “determinant factors”AND “university students”OR “residents”OR "health care workers" AND "Ethiopia"	540
Google Scholar	“blood donation” OR “blood donation practice” AND "factors" OR “associated factors” OR “determinant factors”AND “university students”OR “residents”OR "health care workers" AND "Ethiopia"	691
Other databases		09
Total retrieved articles		1240
Number of included studies		20

Table S3: Study characteristics included in the review and meta-analysis of blood donation practices and associated factors in Ethiopia.

Author	Publication year	Region	Study participant	Method of survey	Sample size	Prevalence
Misganaw C et al.	2014	Addis Ababa	University student	Self-administered	384	23.44
Teklu S et al.	2015	Addis Ababa	Health care worker	Self-administered	295	61.69
Nigatu A & Demissie D.	2014	Oromia	University student	Self-administered	399	23.56
Mulatu K et al.	2017	SNNP	Community	Face to face interview	250	26.40
Gebresilase H et al.	2017	Oromia	University student	Self-administered	360	21.94
Abera B et al.	2017	Amhara	Health care worker	Self-administered	276	42.75
Mekonnen F & Melese S.	2016	Amhara	Community	Face to face interview	387	18.09
Addisu AG et al.	2017	Amhara	Community	Face to face interview	376	10.64
Malako D et al.	2019	SNNP	Health care worker	Self-administered	218	21.56
Beyene GA et al.	2020	Oromia	Community	Face to face interview	410	17.07
Mijena GF et al.	2019	Oromia	Community	Self-administered	383	43.60
Jemberu YK et al.	2016	Amhara	Community	Face to face interview	772	16.06
Melaku M et al.	2016	Amhara	Community	Face to face interview	768	18.36
Melaku M et al.	2018	Amhara	University student	Self-administered	255	12.55
Arage G et al.	2017	Amhara	Health care worker	Self-administered	427	33.26
Urgesa K et al.	2017	Oromia	Community	Face to face interview	845	22.60
Derega B et al.	2015	Oromia	University student	Self-administered	609	18.39
Tadesse T et al.	2018	Other	Health care worker	Self-administered	556	47.84
Tadesse W et al.	2018	Other	University student	Self-administered	339	24.48
Seid T et al.	2017	Other	Health care worker	Self-administered	237	38.82

SNNP, South Nation Nationalities and Peoples, Other, Tigri region and Afar region

Table S4: Sensitivity analysis on blood donation practice and associated factors in Ethiopia

Study omitted	Estimate prevalence	(95%CI)
Misganaw C et al.(2014)	25.96	21.37-30.54
Teklu S et al.(2015)	25.18	20.79-29.57
Nigatu A and Demissie DB (2014)	25.94	21.36-30.54
Mulatu K et al (2017)	25.80	21.26-30.33
Gebresilase HW et al.(2017)	26.03	21.45-30.62
Abera B et al.(2017)	24.96	20.66-29.27
Mekonnen FH and Melese ST(2016)	26.24	21.66-30.83
Addisu AG et al.(2017)	26.63	22.27-31.00
Malako D et al.(2019)	26.05	21.50-30.59
Beyene GA et al.(2020)	26.30	21.72-30.87
Mijena GF et al.(2019)	24.88	20.67-29.09
Jemberu YK et al.(2016)	26.36	21.75-30.97
Melaku M et al.(2016)	26.24	21.57-30.91
Melaku M et al.(2018)	26.53	22.05-31.00
Arage G et al.(2017)	25.43	20.96-29.90
Urgesa K et al.(2017)	26.01	21.30-30.73
Derega B et al.(2015)	26.23	21.60-30.87
Tadesse T et al.(2018)	24.59	20.77-28.41
Tadesse W et al.(2018)	25.90	21.33-30.47
Seid T et al.(2017)	25.17	20.76-29.57
Overall	25.82	21.45-30.19

CI: Confidence Interval

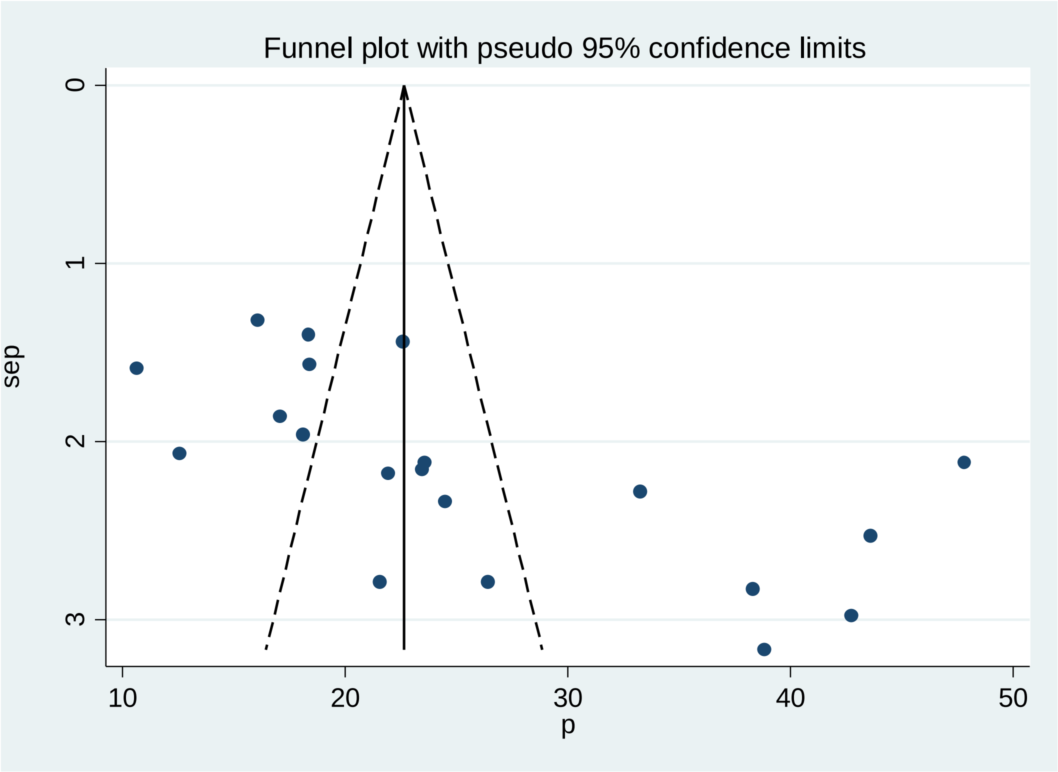

Figure S1: Funnel plot with 95% confidence limits of the pooled prevalence of blood donation practice in Ethiopia.
